# 296. SARS-CoV-2 Illness Severity and Early Hospitalization Outcomes in a Multicenter Prospective Cohort Study Among Veterans

**DOI:** 10.1093/ofid/ofac492.374

**Published:** 2022-12-15

**Authors:** Jennifer M Ross, Jonathan D Sugimoto, Andrew Timmons, Kathryn Moore, Jonathan Adams, Deanna Wilson, Cindy H Liu, Katrina V Deardoff, Anna Korpak, Kyong-Mi Chang, Kelly Cho, Kristina Crothers, Michael Gaziano, Mark Holodniy, Christine M Hunt, Stuart N Isaacs, Barbara E Jones, Elizabeth Le, Javeed Shah, Nicholas L Smith, Jennifer S Lee

**Affiliations:** University of Washington, Seattle, Washington; VA Puget Sound Health Care System, Seattle, Washington; Veterans Health Administration, Seattle, Washington; VA Puget Sound Health Care System, Seattle, Washington; VA Puget Sound Health Care System, Seattle, Washington; Palo Alto VA, Palo Alto, California; VA Puget Sound Healthcare System, Seattle, Washington; VA Puget Sound Healthcare System, Seattle, Washington; VA Puget Sound Health Care System, Seattle, WA, USA, Seattle, Washington; Corporal Michael J. Crescenz VA Medical Center, Philadelphia, Pennsylvania; VA Boston Health Care System, Boston, Massachusetts; VA Puget Sound Health Care System, Seattle, Washington; VA Boston Health Care System, Boston, Massachusetts; Department of Veterans Affairs, Palo Alto, California; Durham Veterans Affairs Health Care System, Durham, North Carolina; Philadelphia VA Medical Center, Philadelphia, Pennsylvania; University of Utah and Salt Lake City VA Healthcare System, Salt Lake City, Utah; VA Palo Alto Health Care System, Palo Alto, California; University of Washington, Seattle, Washington; University of Washington, Seattle, Washington; Palo Alto Veterans Affairs Health Care System, Palo Alto, California

## Abstract

**Background:**

Over 600,000 SARS-CoV-2 infections and 20,000 deaths have occurred among users of the Veterans Health Administration, the US’s largest integrated health care system. We explored early outcomes of SARS-COV-2 infection in Veterans.

**Methods:**

An ongoing, prospective longitudinal cohort study of Veterans ages ≥ 18 enrolled 1,826 participants (29.0% inpatient; 49.1% vaccinated; 68.3% SARS-CoV-2-positive; 85.0% male, mean age = 57.1 years) seeking inpatient or outpatient care after SARS-CoV-2 testing at 15 Department of Veterans Affairs medical centers in July 2020 to February 13, 2022. Using multivariable regression, we estimated relationships of baseline demographic characteristics, COVID-19 vaccination, and clinical history to illness severity and cumulative length of hospital stay within 60 days of study entry. Illness severity was defined by a Veterans Affairs adaptation of the WHO COVID-19 severity scale and included 4 levels (mild, moderate, severe, or death). We derived the Charlson co-morbidity index (CCI) and other baseline characteristics from electronic health data and study questionnaires, and reported qualitative SARS-CoV-2 IgG responses using inpatients’ study-collected blood specimens.

**Results:**

High CCI scores (≥ 5) occurred in 47 (42.7%) vaccinated SARS-CoV-2-positive inpatients and 47 (21.2%) unvaccinated. Severe illness occurred in 17 (15.5%) vaccinated inpatients, 37 (16.7%) unvaccinated inpatients, 4 (0.9%) vaccinated outpatients, and 3 (0.7%) unvaccinated outpatients. Eleven (10%) of 110 vaccinated SARS-CoV-2-positive inpatients died, as did 15 (6.8%) of the 222 unvaccinated. In SARS-CoV-2-positive inpatients, a one-step higher CCI was associated with more severe illness (aOR 1.10, 95% CI 1.01-1.20) and more hospitalization days (aIRR 1.06, 95% CI 1.03-1.10), adjusting for vaccination status. Respectively, 93% of vaccinated and 63% of unvaccinated SARS-CoV-2 positive inpatients with baseline antibody results had an anti-spike IgG response.

**Conclusion:**

In an ongoing longitudinal cohort study of COVID-19 in US Veterans, comorbidity burden was higher among vaccinated than unvaccinated inpatients and was associated with more severe illness and hospitalization days, independent of vaccination status.

**Disclosures:**

**Christine M. Hunt, MD, MPH**, Adaptive Phage Therapeutics: Advisor/Consultant|Akebia: Advisor/Consultant|Galmed: Advisor/Consultant|Otsuka: Advisor/Consultant|Palladio: Advisor/Consultant.

